# Efficacy and safety of tension band wire versus plate for Mayo II olecranon fractures: a systematic review and meta-analysis

**DOI:** 10.1186/s13018-022-03262-7

**Published:** 2022-08-03

**Authors:** Yizhen Jia, Aifeng Liu, Tianci Guo, Jixin Chen, Weijie Yu, Jingbo Zhai

**Affiliations:** 1 Department of Orthopaedic Surgery, First Teaching Hospital of Tianjin University of Traditional Chinese Medicine, Tianjin, China; 2 National Clinical Research Center for Chinese Medicine Acupuncture and Moxibustion, Tianjin, China; 3 Institute of Traditional Chinese Medicine, Tianjin University of Traditional Chinese Medicine, Tianjin, China

**Keywords:** Olecranon fracture, Tension band wire, Plate, Meta-analysis

## Abstract

**Purpose:**

For olecranon fractures, the choice of tension band wire (TBW) or plate fixation has long been controversial. Therefore, this study aimed to evaluate the efficacy and safety of TBW and plate in the treatment of patients with Mayo II olecranon fractures by Meta-analysis.

**Methods:**

PubMed, Embase, Cochrane, the Web of Science, China National Knowledge Infrastructure, Wanfang, and China Biomedical Database were searched for randomized controlled trials (RCTs) and cohort studies (CSs) where TBW was compared with plate for Mayo II olecranon fractures (OF). Subsequently, the data were extracted by two reviewers independently and were analysed via RevMan5.4.1. Besides, mean difference (MD), risk ratio (RR), and 95% confidence intervals (CIs) were calculated. Furthermore, Cochrane Risk of Bias Tool 2.0 and Newcastle–Ottawa Scale were adopted for assessing the risk of bias.

**Results:**

A total of 1RCT and 10 CSs were included, when 449 cases were treated with TBW and 378 with plate. The plate has favourable postoperative long-term (≥ 1 year) functional score in MEPS (MD: − 3.06; 95% CI − 5.50 to 0.62; *P* = 0.01; *I*^2^ = 41%) and Dash score (MD: 2.32; 95% CI 1.91, 2.73; *P* < 0.00001; *I*^2^ = 0%), also carrying fewer complications (RR: 2.13; 95% CI 1.48, 3.08; *P* < 0.0001; *I*^2^ = 58%). Besides, there exists no significant difference in postoperative long-term (≥ 1 year) elbow flexion (MD: − 1.82°; 95% CI − 8.54, 4.90; *P* = 0.60; *I*^2^ = 71%) and extension deficits (MD: 1.52°; 95% CI − 0.38, 3.42; *P* = 0.12; *I*^2^ = 92%). Moreover, TBW is featured with a shorter operation time (MD = − 5.87 min; 95% CI − 7.93, − 3.82; *P* < 0.00001; *I*^2^ = 0) and less intraoperative bleeding (MD: − 5.33 ml; 95% CI − 8.15, − 2.52; *P* = 0.0002; *I*^2^ = 0). In terms of fracture healing time, it is still controversial. Furthermore, the subgroup analysis has revealed that for Mayo IIA OF, the plate has a better outcome in the long-term (≥ 1 year) postoperative MEPS, the Dash score, and the incidence of postoperative complications than TBW, while there is no significant difference in the long-term (≥ 1 year) postoperative elbow motion between two groups.

**Conclusions:**

Plate has better efficacy and safety for Mayo II OF. Considering that few studies are included in the meta-analysis, more high-quality RCTs are still required to confirm these findings.

*PROSPERO registration number*: CRD42022313855.

**Supplementary Information:**

The online version contains supplementary material available at 10.1186/s13018-022-03262-7.

## Introduction

The olecranon fracture (OF) is a common upper limb fracture and often caused by direct violence against the elbow such as a fall or a car accident. Epidemiological investigations have revealed that the incidence of OF represents approximately 10% of upper limb fractures and 18% of forearm fractures [1]. Since the olecranon is a vital part of the elbow joint, its integrity and continuity directly influence the mobility and stability of the elbow joint. Some scholars have found that in all OFs, simple displaced transverse fractures are the most common, among which approximately up to 85% belong to Mayo II OF [2].

At present, the common internal fixation methods for the treatment of the OF include tension band fixation, K-wire/screw tension band fixation, intramedullary nail fixation, and plate fixation [2–4]. However, there is no uniform internal fixation option for Mayo II OF. The AO Fracture Internal Association recommends tension band wire (TBW) fixation for Mayo IIA OF, and plate fixation for Mayo IIB OF [5]. However, in the actual treatment process, as there are considerable differences in fractures, it is difficult to strictly follow the recommended protocol. In such case, the unified treatment standard for Mayo II OF is still in dispute.

As typical treatments for Mayo II OF, TBW and plate have different fixation principles. TBW refers to employing two parallel Kirschner wires to fix the distal and proximal ends of the fracture and using a ‘figure of 8’ loop to convert the extensor forces of the triceps muscle into compressive forces along the articular surface. Comparatively, plate refers to attaching the metal plate to the fracture fragments with screws to bridge the fracture gap and facilitate fracture healing [2, 6].

Some scholars consider that there is no obvious difference in the TBW between treating simple and crushed OF, and thus, it can be used as the gold standard for treating the OF [7, 8]. However, insufficient stability of Kirschner wire causes more complications from TBW fixation (soft tissue stimulation, failure of fixation, etc.), and thus, a more stable plate fixation substitution was proposed by Ren et al. [9]. The latest systematic review of OF treatment found that there was no significant difference in the clinical efficacy of the TBW and plate, but since there were few included studies and the fracture classification was not defined, the conclusions have some limitations [10]. Given the current controversy over the treatment of Mayo II OF, we conducted this systematic review and meta-analysis to investigate the efficacy and safety of TBW versus plate for Mayo II OF.

## Methods

This meta-analysis was performed according to the Preferred Reporting Items for Systematic Reviews and Meta-Analyses (PRISMA) statement. The study was registered in the International Prospective Register of Systematic Reviews (PROSPERO) database (registry no. CRD42022313855).

### Search strategy

A comprehensive search in electronic databases (PubMed, Cochrane, Embase, Web of Science, China National Knowledge Infrastructure, Wanfang Digital Periodicals, and the Chinese Biomedical Literature) was conducted on 28 February 2022 for studies that compared plate with TBW for OF. Here, it should be noted that the search syntax is described in Additional file 1.

### Selection criteria

#### Study design

Randomized controlled trials (RCTs) and retrospective or prospective cohort studies (CSs) were considered for inclusion.

#### Participants

Patients with Mayo II OF were included. Children or patients with pathologic fractures were excluded.

#### Intervention or exposure and control

RCTs or CSs evaluating the efficacy and safety of TBW versus plate for OF were included. A minimal follow-up duration of 6 months was required. Each group should have no less than 10 patients. The surgical procedures were unrestricted.

#### Outcomes

The primary outcome measures included the postoperative Mayo Elbow Performance Score (MEPS), postoperative Disabilities of the Arm, Shoulder and Hand (Dash) score, complications, elbow flexion, and elbow extension deficits. The second outcome measures contained intraoperative bleeding, fracture healing time, and operation time.

### Literature screening

The literature searched from the databases was imported into the Endnote X9 software [11]. After removing the duplicate literature, two reviewers (Y.J and A.L) deleted literature that did not satisfy the inclusion and exclusion criteria based on the title and abstract individually. Besides, the full texts of the remaining literature were reviewed to identify the eligible studies. Disagreement was solved by consensus with a third reviewer (J.Z).

### Data extraction

Two authors (W.Y and J.C) extracted the information independently and finally cross-checked it. The extraction involved the name of the first author, year of publication, country, type of study, intervention, the sample size of two groups, the ratio of males to females, mean age, follow-up time, fracture type, and outcome index. If the information is incomplete, they attempted to contact the author of the original literature.

### Quality assessment of included studies

Two authors (Y.J and T.G) evaluated the literature independently. Cochrane Risk of Bias Tool 2.0 was applied to evaluate the quality of the RCTs [12], and the NOS score was employed to assess the quality of the CSs [13]. The disagreements were resolved by consulting with a third evaluator (J.Z.).

### Statistical analysis

Meta-analysis was performed using RevMan statistical software version 5.4.1 (Cochrane Collaboration). For the dichotomous variables, the risk ratio (RR) and 95% confidence intervals (CIs) were estimated. However, for the continuous variables, the pooled effect was presented as the mean difference (MD) and 95% CIs. *P* < 0.05 indicated a statistically significant difference between the 2 groups. Apart from that, the I^2^ statistic and *P* value were used to evaluate heterogeneity, while a random-effects model was applied when the statistical heterogeneity was high (*P* ≤ 0.10 or *I*^2^ ≥ 50%) [14]. Otherwise, a fixed-effects model was involved (*P* > 0.10 and *I*^2^ < 50%). If possible, subgroup analyses based on age, race, fracture classification, study type, etc., were made when the heterogeneity was high. Publication bias was assessed by the Eggers test using Stata15.0 when the meta-analysis included > 10 studies. Moreover, a narrative description was provided if meta-analysis was infeasible.

## Results

### Literature search

**Fig. 1:**
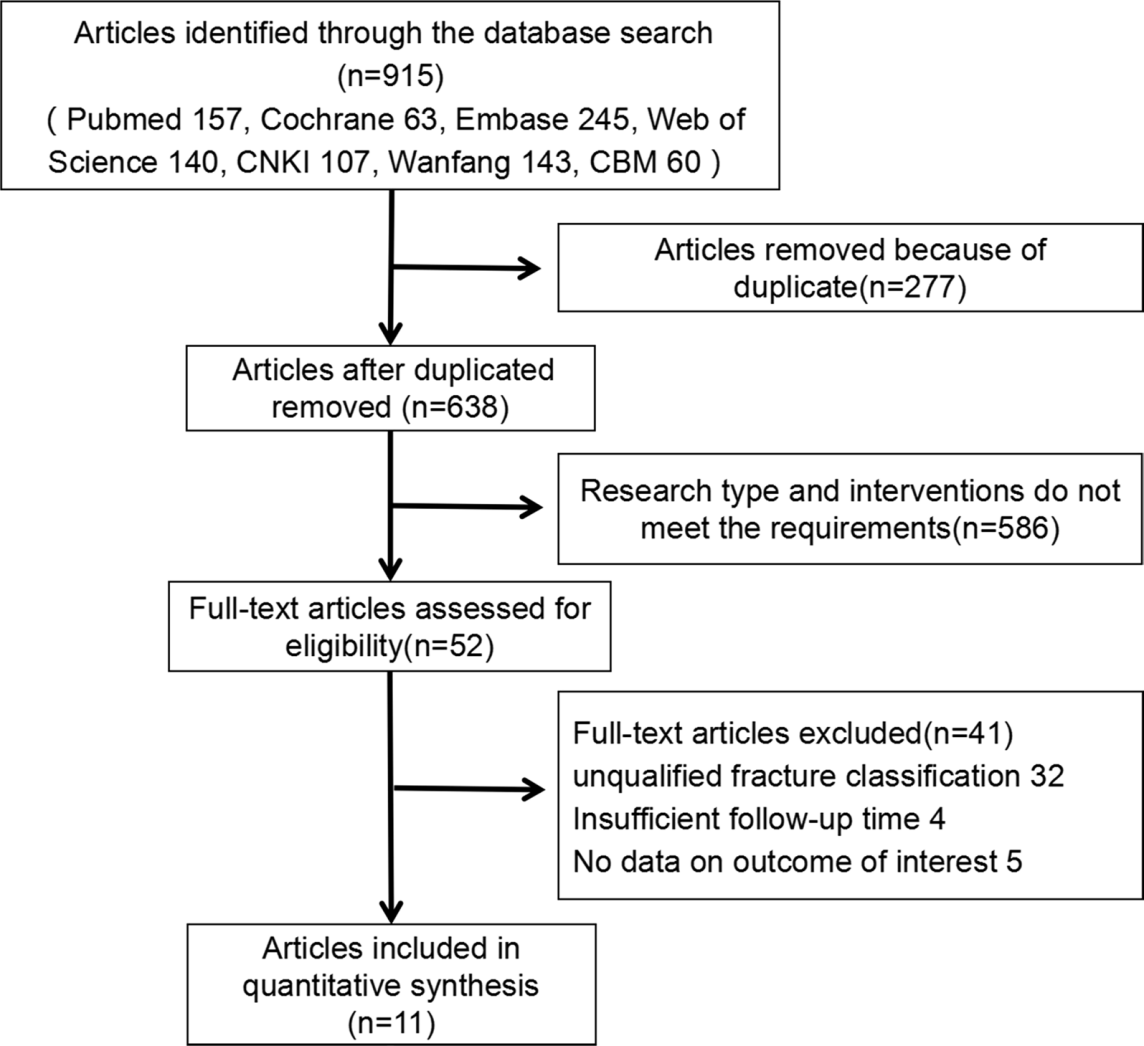
PRISMA flow chart of the literature search

Among the 915 citations identified in the search, we excluded 277 duplicates using Endnote X9 software. A further 586 citations were excluded after the title and abstract screening in line with the selection criteria. Then, 41 articles were further excluded because of unqualified fracture classification, insufficient following time and no data on the outcome of interest. Finally, 11 articles, namely 1 RCT and 10 CSs, were involved [15–24]. Figure 1 presents the flow chart of the literature search and study selection.

### Characteristics and qualifications of included studies

The characteristics of all eleven included studies were summarized and are shown in Table 1. Specifically, 1 RCT and 10 CSs involved 827 patients with OFs (449 patients in TBW, 378 patients in plate). All of them were published between 2014 and 2021. The included RCT had a moderate methodological quality according to the Cochrane Risk of Bias Tool 2.0 [17] (Additional file 2), while the included 10 CSs had moderate-to-high methodological qualities based on the Newcastle–Ottawa Scale, because the total scores were higher than five stars (Table 2).

**Table 1:** Characteristics of included studies

Study	Country	Research type	Number of patients (n)		Sex ratio (male/female)		Age(*X* ± *S*, year)		Follow-up time (month)	Fracture type	Outcomes
Qiu 2021*	China	RCS	TBW	29	TBW	14/15	TBW	33	≥ 15	Mayo IIA, IIB	④⑥⑦
			PL	29	PL	18/11	PL	38			
Çağlar, 2021	Turkey	RCS	TBW	44	TBW	24/20	TBW	40.4 ± 18.1	≥ 36	Mayo IIA	④⑤⑥⑧
			PL	48	PL	27/21	PL	43.7 ± 20.5			
Wang 2021	China	CS	TBW	60	TBW	32/28	TBW	43.32 ± 18.45	12	Mayo IIA	④⑤⑧
			PL	60	PL	34/26	PL	43.72 ± 19.80			
Tan2020	Singapore	RCS	TBW	94	TBW	40/54	TBW	53.1 ± 17.7	12	Mayo IIA	③⑥⑦
			PL	53	PL	19/34	PL	62.6 ± 20.5			
Lu 2020	China	RCS	TBW	42	TBW	25/17	TBW	44.6 ± 15.2	12–48	Mayo IIA, IIB	①②③④⑥⑦
			PL	36	PL	20/16	PL	45.7 ± 17.1			
Powell 2018	England	RCS	TBW	48	TBW	20/28	TBW	57	≥ 28	Mayo IIA	⑥
			PL	16	PL	4/12	PL	60			
Gong 2018	China	RCS	TBW	26	TBW	17/9	TBW	45.3 ± 13.0	18–36	Mayo IIA, IIB	①②③④⑥⑦
			PL	22	PL	15/7	PL	44.1 ± 16.5			
Duckworth 2017	England	RCT	TBW	34	TBW	21/13	TBW	43 ± 16	1.5, 3, 6, 12	Mayo IIA	④⑤⑥⑦
			PL	33	PL	17/16	PL	52 ± 17			
Padilla 2017	Spain	RCS	TBW	26	TBW	6/20	TBW	69	12	Mayo IIA, IIB	⑥⑦⑧
			PL	23	PL	2/21	PL	78			
Schliemann 2014	Germany	CS	TBW	13	TBW	6/7	TBW	38.1	≥ 13	Mayo IIA	⑤⑥⑦⑧
			PL	13	PL	7/6	PL	48.6			
Tarallo 2014	Italy	RCS	TBW	33	TBW	13/20	TBW	51.8 ± 10.1	≥ 12	Mayo IIA, IIB	④⑤⑥⑧
			PL	45	PL	17/28	PL	49.4 ± 12.7			

PL, plate fixation TBW, tension bend wire RCS, retrospective cohort study

① operation time, ② intraoperative bleeding, ③ fracture healing time, ④ Mayo Elbow Performance Score (MEPS), ⑤ Disabilities of the Arm, Shoulder and Hand (Dash) Score, ⑥ complication, ⑦ elbow flexion, ⑧ elbow extension deficit

^*^After propensity score matching analysis

**Table 2:** Newcastle–Ottawa Scale scores for included cohort studies

Study	Selection	Comparability	Outcome	Overall quality score
Çağlar 2021	****	**	***	9
Qiu2021	****	**	**	8
Wang 2021	****	**	**	8
Tan 2020	****	*	**	7
Lu2020	**	**	**	6
Gong 2018	****	**	**	8
Powell 2018	****	*	**	7
Padilla 2017	****	*	***	8
Schliemann 2014	**	*	***	6
Tarallo 2014	****	*	***	8

The total score of this scale is 9. A higher overall score indicates a lower risk of bias; A total score of 5 or less indicates a high risk of bias

^*^Means a score of 1; **Means a score of 2

### Primary outcomes

#### MEPS

**Fig. 2:**
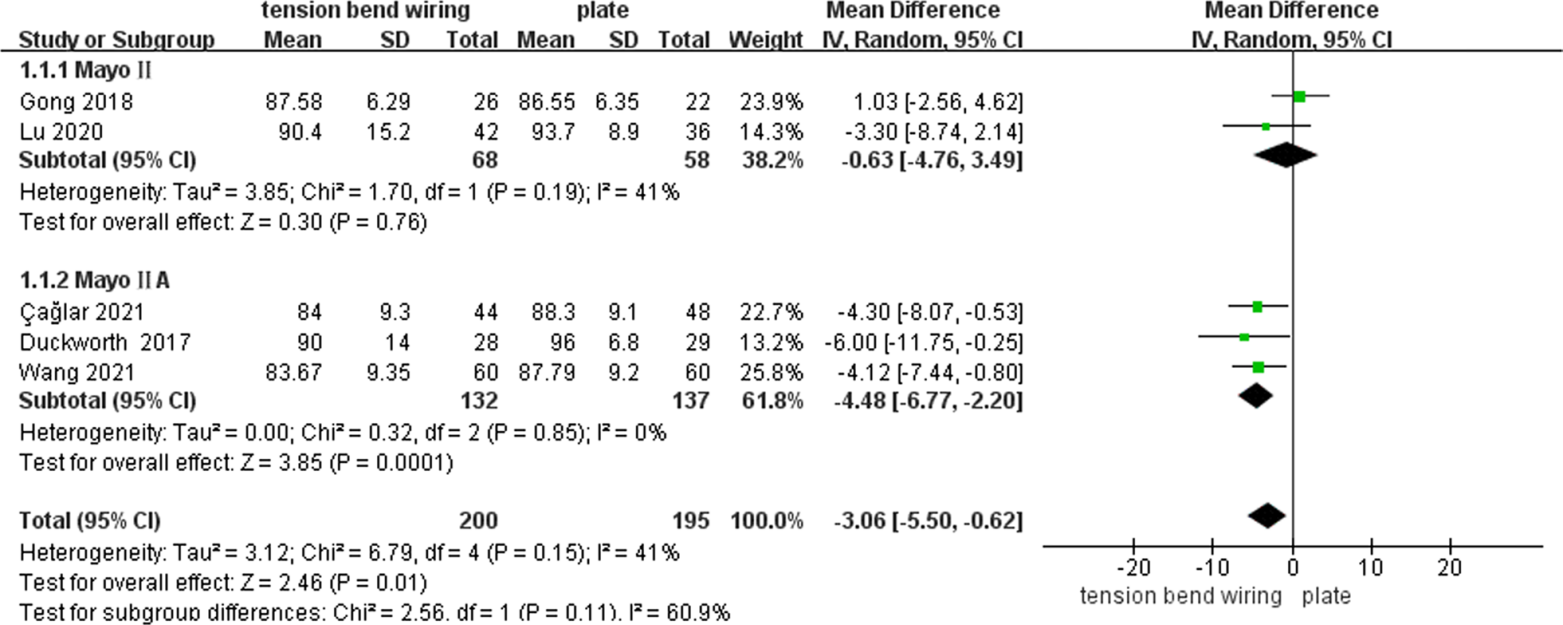
Forest plot of the postoperative MEPS after TBW versus plate for olecranon fractures

Seven studies reported postoperative MEPS (one RCT and six CSs) [15–19, 23, 25]. Two CSs showed no statistically significant differences in the postoperative MEPS between plate and TBW groups of the Mayo II OF (Qiu (MD = − 1.80; 95% CI − 4.68, 1.08; *P* = 0.22) [19] and Tarallo (MD = − 1.80; 95% CI − 6.73 to 3.13; *P* = 0.47) [23]). However, the scores of the plate group were better than those of the TBW group in both studies. They were not included in the meta-analysis for unmentioned measuring time. The other five studies compared the long-term efficacy of 1 year or more, while two CSs compared the efficacy of the Mayo II OF [15, 16]. Besides, two CSs [18, 25] and one RCT [17] compared the efficacy of the Mayo IIA OF. A better long-term MEPS was found in the plate group (MD = − 3.06; 95% CI − 5.50, 0.62; *P* = 0.01; *I*^2^ = *41%*). To further lower the heterogeneity, we performed a subgroup analysis of studies with different fracture classifications. Furthermore, the estimates in the Mayo II OF (MD = − 0.63; 95% CI − 4.76, 3.49; *P* = 0.76; *I*^2^ = 41%) and the Mayo IIA OF (MD = − 4.48; 95% CI − 6.77, − 2.20; *P* = 0.0001; *I*^2^ = 0%) were similar (test for subgroup difference: *P* = 0.11; *I*^2^ = 60.9%) (Fig. 2).

#### Dash scores

**Fig. 3:**
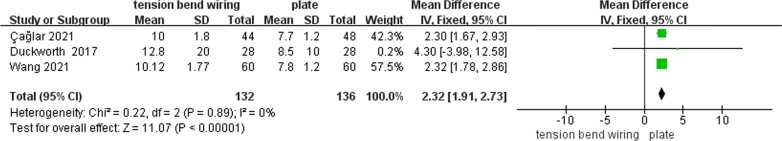
Forest plot of the postoperative Dash score after TBW versus plate for olecranon fractures

Five studies reported postoperative Dash score (one RCT and four CSs) [17, 18, 22, 23, 25], while two CSs examined the Mayo II OF (Tarallo (MD: 1.70; 95% CI − 3.75, 7.15; *P* = 0.54) [23]) and the Mayo IIA OF (Schliemann (MD: 1.50; 95% CI − 7.28, 10.28; *P* = 0.74) [22]), respectively. There existed no statistical difference between both treatment groups, while the scores of the plate group were better than those of the TBW group in both studies. They were not included in the meta-analysis for unmentioned measuring time. In addition, two CSs [18, 25] and one RCT [17] compared the long-term efficacy of the Mayo IIA OF for 1 year or more. Furthermore, the plate group showed better functional scores (MD: 2.32; 95% CI 1.91, 2.73; *P* < 0.00001; *I*^2^ = 0%) (Fig. 3).

#### Elbow flexion

**Fig. 4:**
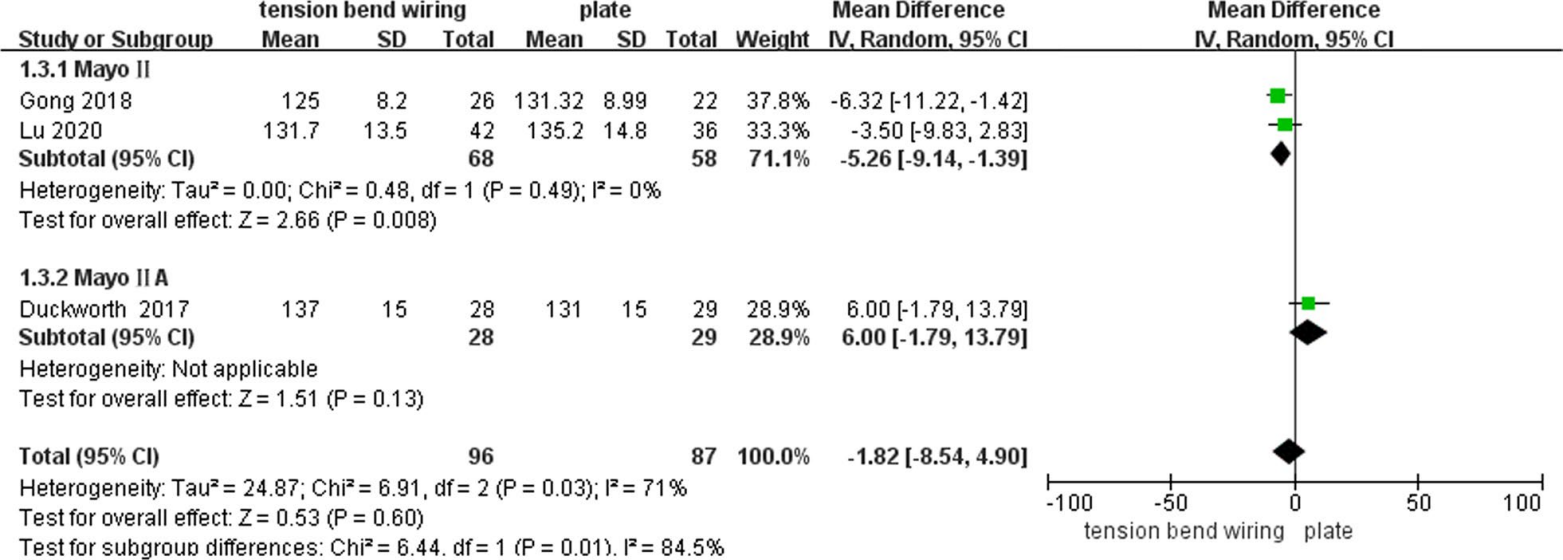
Forest plot of postoperative elbow flexion after TBW versus plate for olecranon fractures

Six studies reported postoperative elbow flexion (one RCT and five CSs) [15–17, 19, 20, 22]. Three CSs examined the Mayo II OF (Qiu (MD: − 1.0°; 95% CI − 6.59, 4.59; *P* = 0.73)) [19] and the Mayo IIA OF (Schliemann (MD: − 3.00°; 95% CI − 7.75, 1.75; *P* = 0.22) [22]; Tan (131° vs 117°; *P* = *0.17*) [20]), respectively. No statistical difference was found between both treatment groups, while the plate group was featured with the better elbow flexion in two studies [19, 22]. They were not included in the meta-analysis for unmentioned measuring time or unavailable data. The other three studies compared the long-term flexion of 1 year or more, while two CSs compared the flexion of the Mayo II OF [15, 16]. Besides, one RCT compared the flexion of the Mayo IIA OF [17]. There was no difference in long-term elbow flexion between both groups (MD: − 1.82°; 95% CI − 8.54, 4.90; *P* = 0.60; *I*^2^ = 71%). To further reduce the heterogeneity, we conducted a subgroup analysis of studies with different fracture classifications. Moreover, the estimates of the Mayo II OF (MD: − 5.26°; 95% CI − 9.14, − 1.39; *P* = 0.008; *I*^2^ = 0) differed from those of the Mayo IIA OF (MD: 6.00°; 95% CI − 1.79, 13.79; *P* = 0.13; *I*^2^ = not applicable) (the test for subgroup difference: *P* = 0.01; *I*^2^ = 84.5%) (Fig. 4).

#### Elbow extension deficit

**Fig. 5:**
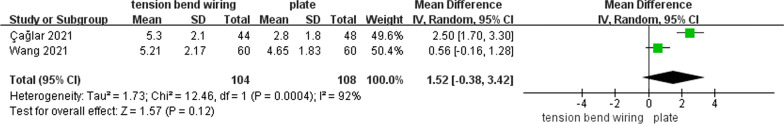
Forest plot of the postoperative elbow extension deficit after TBW versus plate for olecranon fractures

Four CSs reported postoperative elbow extension deficit, while two CSs examined the Mayo II OF (Tarallo (MD: 1.90°; 95% CI − 2.17, 5.97; *P* = 0.36) [23]) and the Mayo IIA OF (Schliemann (MD: − 1.50°; 95% CI − 6.88, 3.88; *P* = 0.58) [22]), respectively. No statistical difference was observed between both treatment groups. They were not included in the meta-analysis for unmentioned measuring time. The other two studies compared the long-term elbow extension deficit of the Mayo IIA OF of 1 year or more [18, 25]. Furthermore, there was no difference in postoperative elbow extension deficit between both groups (MD: 1.52°; 95% CI − 0.38, 3.42; *P* = 0.12; *I*^2^ = 92%) (Fig. 5).

#### Complications

**Fig. 6:**
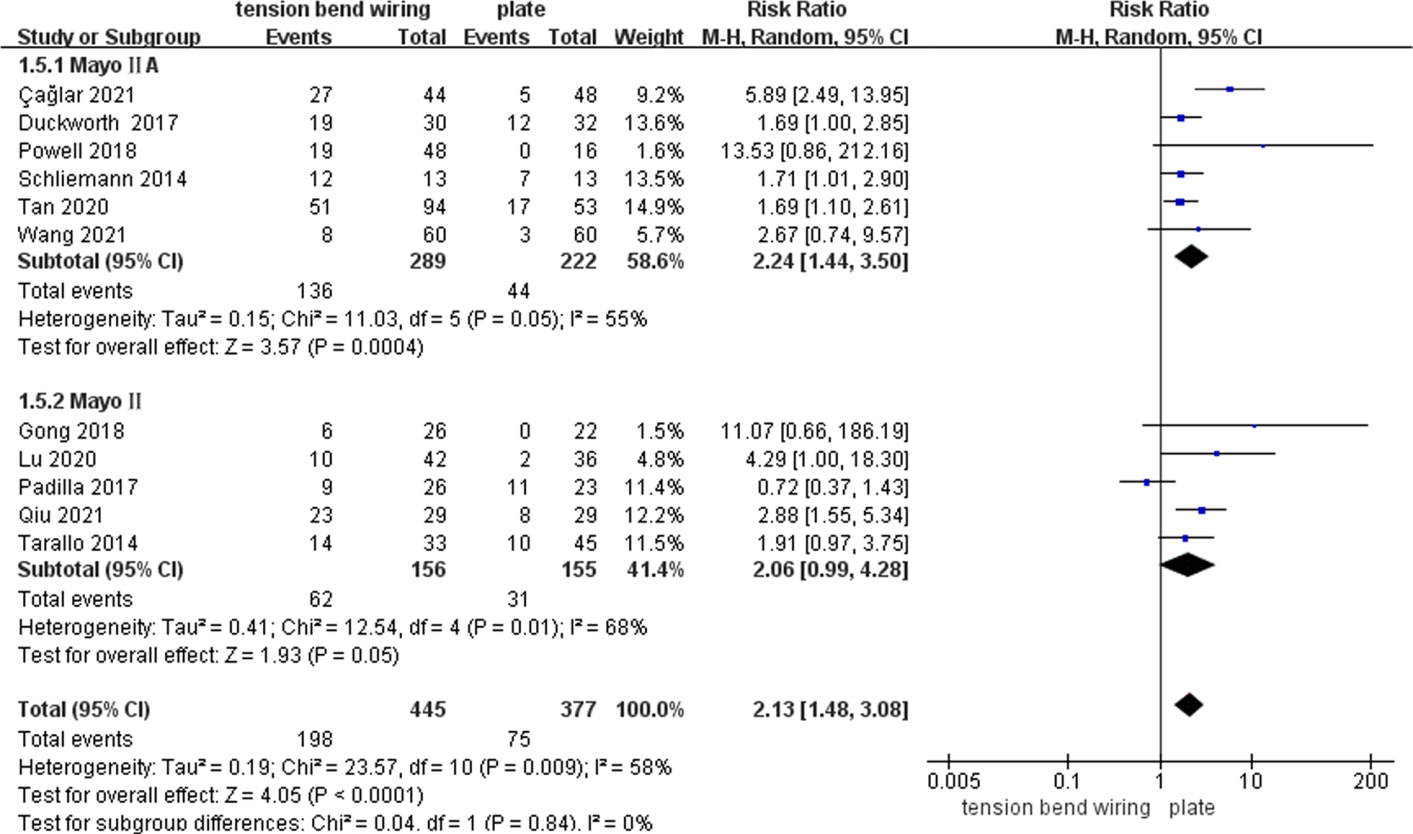
Forest plot of complications after TBW versus plate for olecranon fractures

**Fig. 7:**
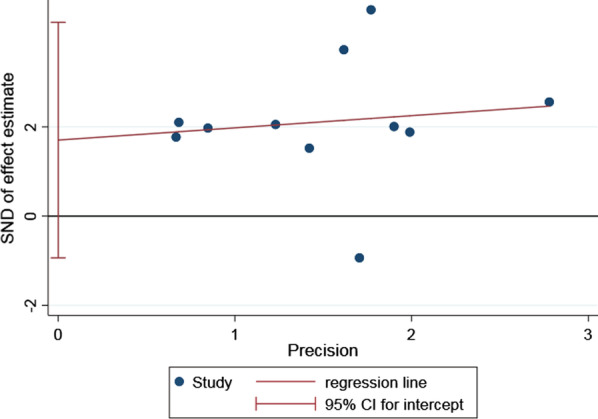
Egger’s test of complications between TBW and plate

The complications were reported in all studies [15–20, 22–26]. Total complications occurred in 44.5% of patients treated with TBW versus 19.9% in the plate group. There was a lower risk of total complications in patients treated with plate (RR 2.13; 95% CI 1.48, 3.08; *P* < 0.0001; *I*^2^ = 58%). To further lower the heterogeneity, we performed a subgroup analysis of studies with different fracture classifications. Besides, the estimates were similar in the Mayo II OF (RR 2.06; 95% CI 0.99, 4.28; *I*^2^ = 68%) and the Mayo IIA OF (RR 2.24; 95% CI 1.44, 3.50; *I*^2^ = 55%) (the test for subgroup difference: *P* = 0.84; *I*^2^ = 0%) (Fig. 6). Egger’s test (*P* = 0.178) presented no publication bias for the complications (Fig. 7).

A further meta-analysis of different complications was conducted. Then, it was found that the risk of implant failure/displacement, implant removal, and implant stimulation in the TBW group was higher than that of the plate group. Moreover, no difference was detected in the risk of other complications. Detailed data are illustrated in Table 3.

**Table 3:** Comparisons of the incidence of complications between TBW and plate

Complication	Number of study	TBW vs plate (RR, 95% CI, *P* value)
Implant removal	9	2.27 [1.41,3.66] *P* = 0.0007
Implant failure/displacement	5	5.72 [1.61,20.35] *P* = 0.007
Implant stimulation	8	2.67 [1.54,4.64] *P* = 0.0005
Infection	8	0.55 [0.23,1.30] *P* = 0.17
Revision	3	1.18 [0.23,6.01] *P* = 0.85
Non-union	3	1.48 [0.42,5.21] *P* = 0.54
Ulnar neuropathy	1	0.45 [0.02,10.73] *P* = 0.62
Radio-ulnar synostosis	2	0.39 [0.04,3.56] *P* = 0.40
Haematoma	1	0.20 [0.01,3.80] *P* = 0.28

### Secondary outcomes

#### Operation time

**Fig. 8:**
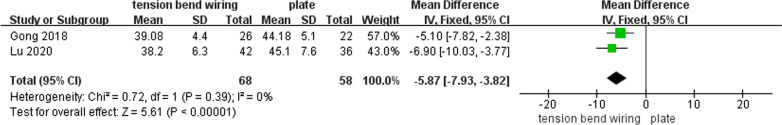
Forest plot of operation time after TBW versus plate for olecranon fractures

Two CSs reported operation time [15, 16], and all examined the Mayo II OF. The TBW group had a shorter operation time (MD = − 5.87 min; 95% CI − 7.93, − 3.82; *P* < 0.00001; *I*^2^ = 0) (Fig. 8).

#### Intraoperative bleeding

**Fig. 9:**
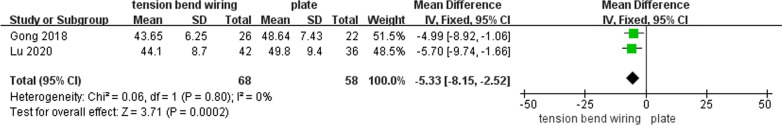
Forest plot of intraoperative bleeding after TBW versus plate for olecranon fractures

Two CSs reported intraoperative bleeding [15, 16], and all examined the Mayo IIOF. The TBW group had less intraoperative bleeding (MD = − 5.33 ml; 95% CI − 8.15 to − 2.52; *P* = 0.0002; *I*^2^ = 0) (Fig. 9).

#### Fracture healing time

**Fig. 10:**
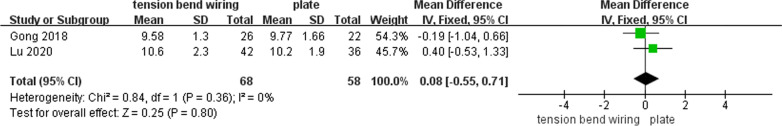
Forest plot of fracture healing time after TBW versus plate for olecranon fractures

Three CSs reported fracture healing time [15, 16, 20]. One CS showed that TBW had a shorter fracture healing time for the Mayo IIA OF (11w VS 15w; *P* < 0.01) [20], while the other two revealed that there existed no difference in fracture healing time between both groups for the Mayo II OF (MD = 0.08w; 95% CI − 0.55 to 0.71; *P* = 0.80; *I*^2^ = 0) (Fig. 10).

## Discussion

In this study, we identified 1 RCT and 10 CSs to evaluate the efficacy and safety of TBW versus plate in the treatment of patients with Mayo II olecranon fractures. According to the results, plate has a favourable postoperative long-term (≥ 1 year) functional score and carries fewer complications. Though no significant differences were observed in postoperative long-term (≥ 1 year) elbow motion, after comprehensive analysis, we believe that plate has a better efficacy and safety for Mayo II olecranon fractures.

As a classic regimen for the OF, TBW has been praised by a large number of clinicians [8, 27, 28]. In virtue TBW does not require extensive dissection of the soft tissue, it maximizes the protection of the blood flow to the fracture site and shortens the operation time. However, due to the lack of stability of Kirschner wire fixation and the differences in the TBW fixation skill of different doctors, it is challenging for the technique to achieve the desired effect, and the TBW fixation is featured with a high risk of internal fixation shifting/failure [29, 30], which was also confirmed in the present study, where the risk of implant failure/displacement in the TBW group was much higher than that in the plate group.

Plate possesses strong and stable fixation properties and can provide long-lasting and effective fracture reduction. Particularly for the olecranon, the plate fixation can better achieve the biological adhesion to the bone, and thus, it is capable of performing effective fixation of various types of fractures [31, 32]. In addition, biomechanical studies also confirmed that plate fixation had less fracture displacement than TBW fixation for the simple OF mode [33, 34]. However, plate fixation also has some deficiencies including large surgical incision, wide soft tissue dissection, and possible intraoperative injury of triceps muscle attachment points [9], which may lead to slow fracture healing, limited elbow joint movement, infection, and other conditions. Nevertheless, in the current research, no supporting evidence was provided. By contrast, plate fixation had fewer complications such as implant failure/displacement and implant stimulation, which is consistent with the conclusions from two previous studies [9, 35], which is sufficient to demonstrate the safety of the plate fixation.

As for efficacy, it was found that for the Mayo II OF, plate fixation obtained a better postoperative long-term (≥ 1 year) functional score. However, in other studies, no difference was found in this respect [9, 35]. The reason may be attributed to the difference to the classification of fractures. Because the classification of fractures has a direct impact on the outcome [19], the comparison of the same type of fracture reduces the heterogeneity and makes the outcome more credible. In terms of elbow motion, no statistical differences were found in elbow flexion and elbow extension deficit. Considering the high heterogeneity of the results, we performed a subgroup analysis on the fracture classification of elbow flexion. A better elbow flexion was found in Mayo II OF subgroup. The difference in outcomes between subgroups may be associated with the presence of type Mayo 2B fractures, for which plate may have a better efficacy. For elbow extension deficit, a subgroup analysis could not be performed with only two studies, but pooled block tended to PF if ignoring the heterogeneity. In conclusion, we believe that plate exerts a better clinical efficacy for Mayo II OF.

For the secondary outcomes, inconsistent conclusions were obtained on the time of fracture healing, which may be related to the fracture subtype, and the presence of type Mayo IIB OF may prolong the fracture healing time of TBW fixation. Although plate fixation showed a worse outcome in terms of the amount of intraoperative bleeding, the difference in the average amount of intraoperative bleeding between the two methods is less than 10 ml, which is not significant in clinical practice. Concerning the operation time, plate takes more time due to the complexity of the operation. Besides, the biggest deficiency of plate fixation lies in the high cost of surgery, which will bring greater economic burden to patients. This is also the main reason why most clinicians prefer TBW fixation. However, the study of Andrew D. Duckworth and A. J. Powell demonstrated that the cost of the TBW fixation was close to or even beyond that of the plate fixation because of the higher rate of revision [17, 26], indicating that the strong and reliable fixation is more significant for clinical outcomes. In addition, Edward M. DelSole discovered that the one-third tubular construct can achieve the same clinical efficacy as locking plate and reduce the cost of approximately $1263.5 [36], implying that choosing the right plate can also decrease the economic pressure of patients to some extent.

The most interesting finding of this study was the better long-term (≥ 1 year) functional scores in both the postoperative MEPS and the Dash score in the plate group for the Mayo IIA OF, which indicated that plate fixation may have better long-term (≥ 1 year) efficacy for the Mayo IIA OF, showing no difference in Ren Yiming’s study [9]. Furthermore, no significant difference is observed in elbow flexion and extension deficits for the Mayo IIA OF. As for complications, the Mayo IIA OF displayed a similar outcome to the Mayo II OF, and both of them had a lower risk of complication rate, demonstrating the advantage of plate over TBW in the treatment of the Mayo IIA OF.

### Limitations

Firstly, the studies included in this study are mainly retrospective cohorts, with certain recall bias. Secondly, there are no independent studies of Mayo IIB fractures. Further analysis of such subtypes is impossible. Thirdly, in this paper, only the TBW and plate fixation are compared, and the results are not applicable to other modified tension band fixation schemes. Fourthly, the results should be interpreted cautiously due to the small number and heterogeneity of the included studies.

## Conclusion

Based on the results of our study, plate fixation has better long-term (≥ 1 year) postoperative MEPS and Dash scores in the Mayo II OF and features a lower risk of complications. Besides, the elbow motion is not significantly different between TBW and plate. Moreover, TBW costs a shorter operation time and less intraoperative bleeding. However, as for the fracture healing time, the results are still controversial between the two groups. Furthermore, for the Mayo IIA OF, the same conclusion in the functional score, elbow motion, and complication can be obtained.

In brief, plate has better efficacy and safety than TBW for Mayo II OF. More high-quality RCTs are still required to confirm the present findings.

## Supplementary Information

**Additional file 1**. Search Strategy (Pubmed).

**Additional file 2**. **Table S1.** Revised Cochrane risk of bias tool for randomized controlled trial (RoB2.0). **Figure S1.** Risk of bias summary in 1 RCT.

## Abbreviations

TBW: Tension band wire
OF: Olecranon fracture
MEPS: Mayo Elbow Performance Score
Dash: Disabilities of the Arm, Shoulder and Hand
CIs: Confidence interval
RR: Risk ratio
MD: Mean difference
