# Supplementary material for: Efficacy and safety of tension band wire versus plate for Mayo II olecranon fractures: a systematic review and meta-analysis
Source: J Orthop Surg Res. 2022 Aug 3;17:373. doi: 10.1186/s13018-022-03262-7 (PMC9351198; doi:10.1186/s13018-022-03262-7)
Supplement: Supplementary file 1 — Additional file 1. Search Strategy (Pubmed). [file 13018_2022_3262_MOESM1_ESM.docx]

**SUPPLEMENTARY 1**

**Search Strategy**

PUBMED

#1 Olecranon Process/

#2 Olecranon*[text word]

#3 #1 OR #2

#4 Fractures, Bone/OR Fracture Fixation/ OR Fracture Healing

#5 fractur*[text word]

#6 #4 OR #5

#7 #3 AND #6

#8 Randomized controlled trial [Publication Type]

#9 Controlled clinical trial [Publication Type]

#10 Cohort clinical trial [Publication Type]

#11 randomized [Title/Abstract]

#12 placebo [Title/Abstract]

#13 randomly [Title/Abstract]

#14 trial [Title/Abstract]

#15 groups [Title/Abstract]

#16 #8 OR #9 OR #10 OR #11 OR #12 OR #13 OR #14 OR #15

#17 (Animals[Mesh Terms]) NOT (Humans[Mesh Terms])

#18 #16 NOT #17

#19 #7 AND #18
