# Supplementary material for: Efficacy and safety of tension band wire versus plate for Mayo II olecranon fractures: a systematic review and meta-analysis
Source: J Orthop Surg Res. 2022 Aug 3;17:373. doi: 10.1186/s13018-022-03262-7 (PMC9351198; doi:10.1186/s13018-022-03262-7)
Supplement: Supplementary file 2 — Additional file 2. Table S1. Revised Cochrane risk of bias tool for randomized controlled trial (RoB2.0). Figure S1. Risk of bias summary in 1 RCT. [file 13018_2022_3262_MOESM2_ESM.docx]

**Supplementary material 2.**

**Table S1. Revised Cochrane risk of bias tool for randomized controlled trial (RoB2.0)**

| **Unique ID** | Duckworth2017 | **Study ID** | NCT01391936 | **Assessor** | Jia and Guo |
| --- | --- | --- | --- | --- | --- |
| **Ref or Label** | J Bone Joint Surg Am. 2017;99:1261-73 | **Aim** | assignment to intervention (the 'intention-to-treat' effect) |  |  |
| **Experimental** | TBW | **Comparator** | PF | **Source** |  |
| **Outcome** | Dash,Mayo,elbow flexion,complication | **Results** | MD(0),OR(1.00) | **Weight** | 1 |
| **Domain** | **Signalling question** | | | **Response** | **Comments** |
| **Bias arising from the randomization process** | 1.1 Was the allocation sequence random? | | | Y | After providing informed consent, patients were randomized to undergo either TBW or platefixation. This was performed by block randomization (n = 4) using sequential closed opaque envelopes, which were prepared by our statistician and contained a card detailing to which of the 2 groups (TBW or plate) the patient had been randomized. Randomization was on  a 1:1 basis. |
|  | 1.2 Was the allocation sequence concealed until participants were enrolled and assigned to interventions? | | | Y |  |
|  | 1.3 Did baseline differences between intervention groups suggest a problem with the randomization process? | | | Y | The baseline demographic and fracture characteristics of the 2 treatment groups are shown in Table II. Patients in the TBW group were younger (43 versus 52 years; p = 0.028), but all other characteristics were comparable. |
|  | **Risk of bias judgement** | | | **Some concerns** |  |
| **Bias due to deviations from intended interventions** | 2.1.Were participants aware of their assigned intervention during the trial? | | | N | This is a single-blinded, prospective randomized trial.The Surgery can't be blinded to the doctor. |
|  | 2.2.Were carers and people delivering the interventions aware of participants' assigned intervention during the trial? | | | Y |  |
|  | 2.3. If Y/PY/NI to 2.1 or 2.2: Were there deviations from the intended intervention that arose because of the experimental context? | | | PY | Because of the proximal nature of the fracture,1 patient in the plate group underwent TBW fixation, and because of the unexpected comminution of the fracture, 1 patient in the TBW group underwent plate fixation. |
|  | 2.4 If Y/PY to 2.3: Were these deviations likely to have affected the outcome? | | | N | After grouping, the number of patients who changed the treatment plan was the same in both groups, all are one. |
|  | 2.5. If Y/PY/NI to 2.4: Were these deviations from intended intervention balanced between groups? | | | NA |  |
|  | 2.6 Was an appropriate analysis used to estimate the effect of assignment to intervention? | | | NI | We didn't find relevant content. |
|  | 2.7 If N/PN/NI to 2.6: Was there potential for a substantial impact (on the result) of the failure to analyse participants in the group to which they were randomized? | | | PN | The number of patients who changed the treatment plan was the same in both groups, all are one. |
|  | **Risk of bias judgement** | | | **Some concerns** |  |
| **Bias due to missing outcome data** | 3.1 Were data for this outcome available for all, or nearly all, participants randomized? | | | PN | There's a certain number of missing interviews. |
|  | 3.2 If N/PN/NI to 3.1: Is there evidence that result was not biased by missing outcome data? | | | PN | We didn't find relative content. |
|  | 3.3 If N/PN to 3.2: Could missingness in the outcome depend on its true value? | | | PN | The internal fixation of the fracture itself rarely results in the missingness. |
|  | 3.4 If Y/PY/NI to 3.3: Is it likely that missingness in the outcome depended on its true value? | | | NA |  |
|  | **Risk of bias judgement** | | | **Low** |  |
| **Bias in measurement of the outcome** | 4.1 Was the method of measuring the outcome inappropriate? | | | N | The method of measuring the outcome are appropriate. |
|  | 4.2 Could measurement or ascertainment of the outcome have differed between intervention groups? | | | N | The measurement of the two groups are consistent. |
|  | 4.3 Were outcome assessors aware of the intervention received by study participants? | | | N | A full clinical outcome assessment was completed by a blinded research  physiotherapist or research fellow not involved in the patient’s management. |
|  | 4.4 If Y/PY/NI to 4.3: Could assessment of the outcome have been influenced by knowledge of intervention received? | | | NA |  |
|  | 4.5 If Y/PY/NI to 4.4: Is it likely that assessment of the outcome was influenced by knowledge of intervention received? | | | NA |  |
|  | **Risk of bias judgement** | | | **Low** |  |
| **Bias in selection of the reported result** | 5.1 Were the data that produced this result analysed in accordance with a pre-specified analysis plan that was finalized before unblinded outcome data were available for analysis? | | | Y |  |
|  | 5.2 ... multiple eligible outcome measurements (e.g. scales, definitions, time points) within the outcome domain? | | | PN |  |
|  | 5.3 ... multiple eligible analyses of the data? | | | N | There is only one possible way to analyze the outcome. |
|  | **Risk of bias judgement** | | | **Low** |  |
| **Overall bias** | **Risk of bias judgement** | | | **Some concerns** |  |


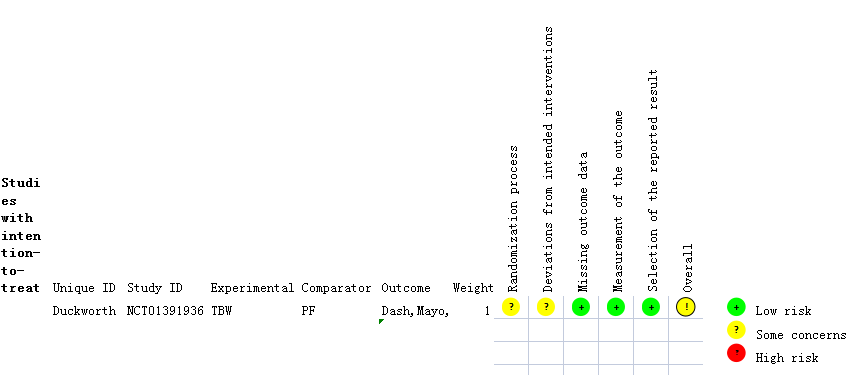


**Figure S1.Risk of bias summary in 1 RCT**
